# Role of ATG7-dependent non-autophagic pathway in angiogenesis

**DOI:** 10.3389/fphar.2023.1266311

**Published:** 2024-01-10

**Authors:** Jinxiang Chen, Yu Liang, Shaorun Hu, Jun Jiang, Min Zeng, Mao Luo

**Affiliations:** ^1^ Basic Medicine Research Innovation Center for Cardiometabolic Diseases, Ministry of Education, Southwest Medical University, Luzhou, Sichuan, China; ^2^ School of Pharmacy, Southwest Medical University, Luzhou, Sichuan, China; ^3^ Key Laboratory of Medical Electrophysiology, Ministry of Education and Medical Electrophysiological Key Laboratory of Sichuan Province, Institute of Cardiovascular Research, Southwest Medical University, Luzhou, Sichuan, China; ^4^ The Affiliated Traditional Chinese Medicine Hospital, Southwest Medical University, Luzhou, Sichuan, China; ^5^ Department of General Surgery (Thyroid Surgery), The Affiliated Hospital, Southwest Medical University, Luzhou, Sichuan, China; ^6^ Metabolic Vascular Diseases Key Laboratory of Sichuan Province, Luzhou, Sichuan, China; ^7^ Department of Pharmacy, The Affiliated Hospital, Southwest Medical University, Luzhou, Sichuan, China

**Keywords:** ATG7, angiogenesis, non-autophagic pathway, NF-κB, STAT1

## Abstract

ATG7, one of the core proteins of autophagy, plays an important role in various biological processes, including the regulation of autophagy. While clear that autophagy drives angiogenesis, the role of ATG7 in angiogenesis remains less defined. Several studies have linked ATG7 with angiogenesis, which has long been underappreciated. The knockdown of ATG7 gene in cerebrovascular development leads to angiogenesis defects. In addition, specific knockout of ATG7 in endothelial cells results in abnormal development of neovascularization. Notably, the autophagy pathway is not necessary for ATG7 regulation of angiogenesis, while the ATG7-dependent non-autophagic pathway plays a critical role in the regulation of neovascularization. In order to gain a better understanding of the non-autophagic pathway-mediated biological functions of the autophagy-associated protein ATG7 and to bring attention to this expanding but understudied research area, this article reviews recent developments in the ATG7-dependent non-autophagic pathways regulating angiogenesis.

## 1 Introduction

Autophagy refers to the regulation of stress-induced lysosomal degradation pathway through a series of autophagy-related genes (ATGs), which plays an important role in stabilizing the intracellular environment (Levine and Kroemer, 2019; Ligeon et al., 2021). Autophagy is separated into macroautophagy, microautophagy, and molecular chaperon-mediated autophagy depending on how cellular contents are transported to the lysosomes (Cui et al., 2021; Wang et al., 2022). So far, more than 40 ATGs have been identified to participate in orchestrating the process of macroautophagy (Mizushima and Levine, 2020). Among them, autophagy-related 7 (ATG7) has ubiquitin E1-like ligase activity, which can facilitate the lipidation of microtubule-associated protein 1 light chain 3 (LC3) and the conjugation of ATG5 and ATG12 (Grant, 2016; Mathew et al., 2021). These two ubiquitin-like conjugation systems directly promote the elongation of the autophagosomal membranes, thereby facilitating the maturation of the autophagosomes. The majority of current research on ATG7 focuses on how it controls the autophagy pathway’s advancement and its function in autophagy. Although it is generally believed that ATG7 is an essential part of autophagosome formation, there is evidence that ATG7 is not necessary for autophagy to proceed (Nishida et al., 2009; Arakawa et al., 2017; Urbańska and Orzechowski, 2021). Even without ATG7, autophagy is still possible, but other processes, including as pathogen defense, cell cycle progression, and vascular biological functions, may be hampered (Lee et al., 2012; Ye et al., 2014; Zhuang et al., 2017).

In recent years, a growing body of evidence has indicated that ATG7 may have key functions in the control of angiogenic and vasculogenic processes. Of note, no studies have yet been published that the autophagy-related protein ATG7 is dependent on autophagy mechanisms in regulating neovascularization, but ATG7 can regulate angiogenesis through non-autophagic pathways. In light of this, this review collates the existing evidence supporting a role for ATG7-dependent non-autophagic pathways in angiogenesis. The potential of ATG7 as a novel drug molecular target for treating angiogenesis-related diseases has also been prospected. Furthermore, these findings may also provide a specific perspective for understanding the non-autophagic biological functions of autophagy proteins.

## 2 Structure and function of ATG7

**FIGURE 1:**
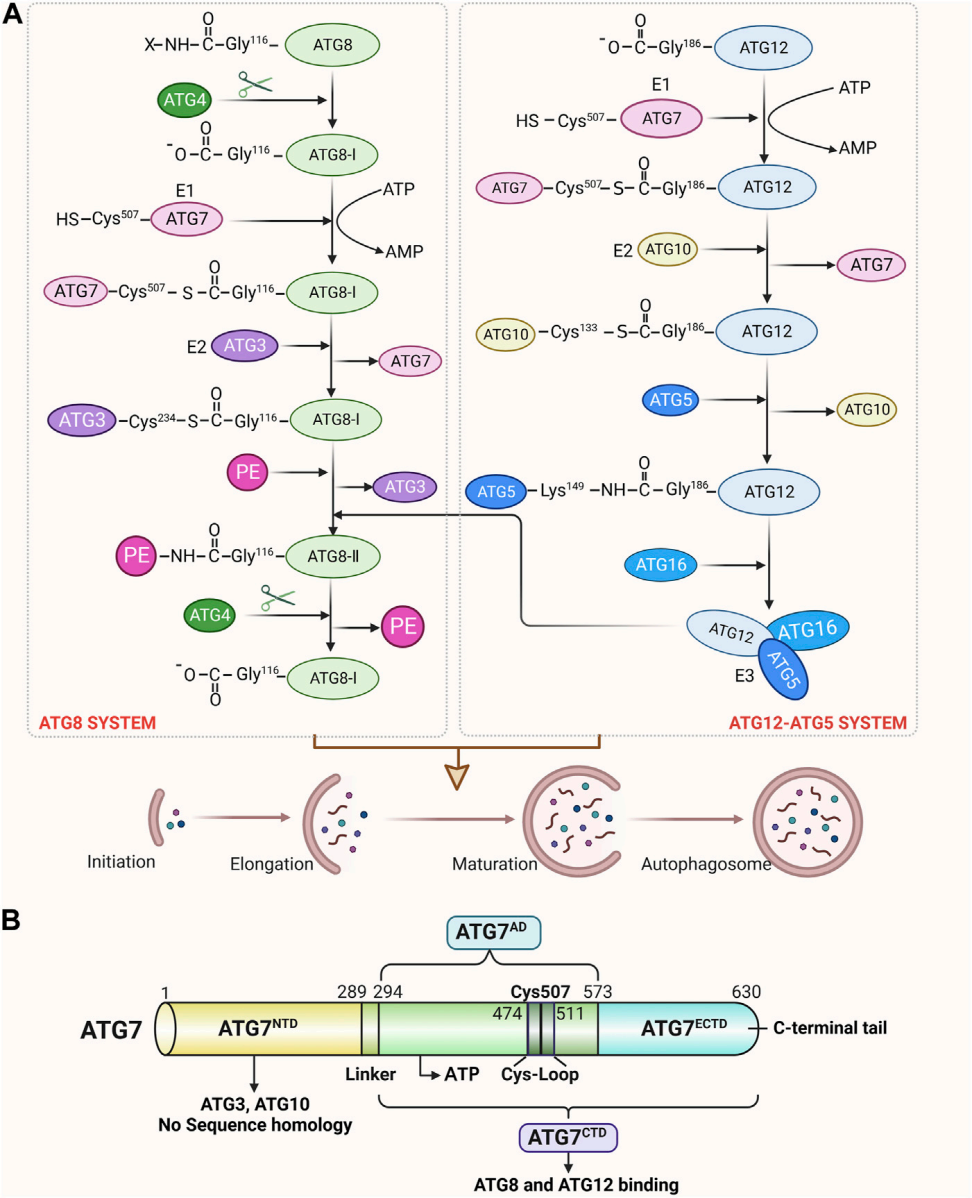
Structure of ATG7 and its role in autophagy. **(A)** Schematic view of ATG7 participating in ATG12 and ATG8 conjugation cascades. In the ATG12-ATG5 system, the C-terminal carboxyl group of ATG12 is activated by the E1 enzyme ATG7 in an ATP-dependent manner. After activation, ATG12 is transferred to the E2 enzyme ATG10 and finally forms an isopeptide bond with the lysine residue in ATG5. ATG5 interacts further with ATG16 and forms a multimeric complex. In the ATG8 system, ATG8 is first cleaved at the C-terminus by the ATG4 protease to expose the glycine residue. This exposed glycine forms a thioester bond with ATG7 in an ATP-dependent manner. Activated ATG8-I is then transferred to the E2 enzyme ATG3 and finally conjugated to PE via an amide bond. ATG12∼ATG5∼ATG16 conjugate acts as an E3-like ligase system to enhance the E2 activity of ATG3 and facilitate the conjugation of ATG8-I with PE. Moreover, ATG4 cleaves ATG8-II∼PE to liberate free ATG8-I from membranes, which can also regulate autophagosome formation. **(B)** Schematic of ATG7 showing positions of NTD and CTD. The N-terminal domain (NTD; amino acids 1–289), which is capable of binding either ATG10 or ATG3, does not display significant homology with other E1s. The C-terminal domain (CTD) is comprised of the homodimeric adenylation domain (AD; amino acids 294–572) that is conserved among all E1s and the extreme C-terminal ATG7-specific domain (ECTD; amino acids 573–630). CTD is responsible for ATG8 and ATG12 binding, and AD possesses the ATP-binding site and a cysteine loop (CL). Residues 474-511 contain the catalytic Cys507 and correspond to a CL. A short linker connects the ATG7^NTD^ and ATG7^CTD^.

The autophagy process is carried out by a series of ATGs, including ATG7, which is in charge of taking part in the development of the autophagosome and the binding system for autophagy as well as playing a crucial regulatory role in the early stages of autophagy. The ATG7 gene encodes a protein of 630 amino acids, which has two main domains: an N-terminal domain (NTD; amino acids 1–289) and a C-terminal domain (CTD; amino acids 294–630) (Kaiser et al., 2013). The CTD is composed of an extreme C-terminal ATG7-specific domain (ECTD; amino acids 573–630) and a homodimeric adenylation domain (AD; amino acids 294–572), which assumes all the functions of the E1 ubiquitination enzyme, whereas the NTD does not share significant homology with the conventional E1 ubiquitination enzyme (Hong et al., 2011; Noda et al., 2011; Taherbhoy et al., 2011). These ATG7 domains can stimulate the covalent attachment of ATG5 to ATG12 and phosphatidylethanolamine (PE) to ATG8, which can then encourage the autophagosome’s transformation. In the ATG12-ATG5 conjugation system, ATG12 is first activated through the formation of a thioester bond between the C-terminal Gly186 of ATG12 and the Cys507 of ATG7 (an E1-like activating enzyme) in an ATP-dependent manner (Geng and Klionsky, 2008; Karow et al., 2020), then transfers to the Cys133 of ATG10 (an E2-like conjugation enzyme) and finally attaches to the Lys149 of ATG5, forming an isopeptide-bonded ATG12∼ATG5 conjugate (Kaiser et al., 2012; Romanov et al., 2012). When combined with ATG16, the ATG12∼ATG5 conjugate forms a substantial protein complex that functions as an E3-like ligase system to promote the conjugation of ATG8 with PE (Martens and Fracchiolla, 2020; Qiu et al., 2020; Munzel et al., 2021). In the second ubiquitin-like conjugation system, ATG8 is first cleaved by the ATG4 protease, which exposes C-terminal glycine residues and is converted into ATG8-I (Negrete-Hurtado et al., 2020). ATG8-I is then attacked by the E1-like activating enzyme ATG7 via a thioester bond between the Gly116 of ATG8-I and the Cys507 of ATG7 (Noda et al., 2011), which is subsequently transthiolated to the Cys234 of ATG3 (an E2-like conjugation enzyme) to form a thioester-linked ATG3∼ATG8-I intermediate (Noda et al., 2011; Yamaguchi et al., 2018). The autophagy cascade is eventually started when Gly116 of ATG8-I links with PE through an amide bond to create ATG8-II∼PE (Collier et al., 2021). Unlike the ATG12∼ATG5 conjugate, the lipidation of ATG8 is reversible. ATG8-II∼PE can be cleaved by ATG4 to release free ATG8-I, which is also essential for autophagosome biogenesis (Ni et al., 2015). ATG7 is the first E1-like activating enzyme to be proven effective in recognizing and activating two different ubiquitin systems that directly promote the elongation or formation of autophagosomal membranes, which can lay the foundation for the fusion of autophagosomes with lysosomes. Here, the structure and function of ATG7 and its involved in the autophagy binding system and autophagosome formation are shown in Figure 1.

Most previous studies related to ATG7 focused exclusively on autophagy, and thus the non-autophagic roles of ATG7 are often overlooked. Various analyses of ATGs have revealed many new interacting components, including those that affect other cellular pathways. This has prompted the question of whether the ATG7, which is associated to autophagy, has a more extensive function that goes beyond autophagy. Several studies have found that the key role played by ATG7 in the regulation of vascular functions, including angiogenesis and inflammatory responses, is dependent on autophagy-independent pathways. For example, ATG7-dependent non-autophagic pathways play a non-negligible role in cerebral angiogenesis (Zhuang et al., 2017; He et al., 2019). Knockdown of ATG7 in mouse brain endothelial cells can significantly improve acute brain injury and brain inflammation induced by ischemia-reperfusion (Wang et al., 2018). These results open up fresh avenues for investigating ATG7’s role in vascular biology, although the underlying mechanisms are still largely unknown.

## 3 ATG7 regulates angiogenesis via non-autophagic pathways

The non-autophagic roles of autophagy proteins have gradually attracted attention. Indeed, growing evidence has suggested the non-autophagic functions of autophagy proteins. ATG5 is a gene product required for the formation of autophagosomes. Yousefi et al. (2006) reported that ATG5, in addition to the promotion of autophagy, enhances susceptibility towards apoptotic stimuli. Casp-mediated cleavage abrogates the autophagic function of Beclin-1, and generates a Beclin-1-C fragment to enhance apoptosis (Wirawan et al., 2010). ATG16L1 was also found to participate in hormone secretion from PC12 cells independently of autophagic activity (Ishibashi et al., 2012). Autophagy complexes have also been found to exert similar, non-autophagic effects. ATG5-ATG12 conjugate negatively regulates the type I IFN production pathway by direct association with the retinoic acid-inducible gene I (RIG-I) and IFN-beta promoter stimulator 1 (IPS-1) through the caspase recruitment domains (CARDs) (Jounai et al., 2007). *Brucella* selectively co-opts autophagy-initiation complexes ULK1, beclin 1, ATG14L to subvert host clearance and promote infection (Starr et al., 2012). Also, IFNγ exerts antiviral activity by inhibiting the formation of the membranous cytoplasmic MNV replication through ATG5-ATG12/ATG16L1 (Hwang et al., 2012). Here, the non-autophagic roles of autophagy proteins are shown in Supplementary Table S1, which shows that transcription, cell survival and apoptosis, cellular transport, protein secretion, cell signaling and membrane reorganization, and all of these non-autophagic functions are closely related to autophagy proteins. We begin by bringing together information on the non-autophagic roles of the ATGs and then highlight the roles of ATG7 in angiogenesis.

There has been an increased interest in the role of ATG7 in angiogenesis. Angiogenesis is the process of forming new blood vessels in an orderly manner (Wang et al., 2021), which involves a variety of cells and cytokines, and the related molecular mechanisms are numerous and diverse (He et al., 2021). Dysregulated angiogenesis can cause various pathologies, including ischemic cardiovascular diseases, cerebrovascular diseases, cancers, inflammatory diseases, etc. Therefore, a deeper comprehension of the precise molecular mechanism by which ATG7 controls angiogenesis may result in the development of further therapeutic methods for the prevention or more potent treatment of angiogenesis disorders. There are currently many studies focusing on the role of autophagy in angiogenesis, and it has been found that ATG7 can act as an autophagy marker and participate in autophagy-mediated angiogenesis. For example, miR-210-3p was found to suppress autophagy and inflammatory activation by targeting ATG7, thereby inhibiting angiogenesis (Li G. et al., 2021a). Circ-ADAM9 upregulated PTEN and ATG7 in interaction with mir-20a-5p, and that inhibited the phosphorylation of AKT and mTOR to aggravate autophagy and apoptosis of EPCs, thus regulating angiogenesis in diabetic pathological conditions (Tian et al., 2020). Notably, several studies have reported the regulation of angiogenesis by ATG7-dependent non-autophagy pathways. This review focuses on the non-autophagic functions of the autophagy-related protein ATG7 in angiogenesis. Supplementary Table S2 indicates that ATG7 participates in autophagy- and non-autophagic-mediated angiogenesis.

### 3.1 ATG7 regulates angiogenesis through NF-κB activation

#### 3.1.1 ATG7 promotes angiogenesis by regulating IL-6 through NF-KB activation

An essential stage in the development of the central nervous system (CNS) is the establishment of the cerebrovascular system, and cerebral angiogenesis plays a role in the functional recovery of brain injuries and ischemic strokes (IS) (Fei et al., 2021; Rustenhoven et al., 2021). A relationship between ATG7 and the cerebrovascular system *in vivo* was first observed in 2017 when ATG7-deficient mice were observed to have significantly reduced brain microvessel density (Zhuang et al., 2017). Upon knockdown of ATG7 in human brain microvascular endothelial cells (HBMECs), the expression of interleukin-6 (IL-6) was found to be downregulated, while the expression of vascular endothelial growth factor (VEGF), a factor with pro-angiogenic activity, was not significantly changed. The addition of exogenous IL-6 was also shown to effectively restore impaired angiogenesis and cell migration induced by ATG7 downregulation, indicating that ATG7 deficiency attenuates angiogenesis in HBMECs by reducing IL-6 expression. Further results revealed that ATG7 mediates nuclear factor-kappa B (NF-κB) at the transcriptional level to regulate the expression of IL-6. Subsequently, the nuclear localization of the RelA (p65), a subunit of the NF-κB transcriptional complex, was analyzed to assess NF-κB activation in ATG7-silenced HBMECs. The results showed that nuclear localization of p65 was significantly reduced after ATG7 knockdown, and betulinic acid (an NF-κB agonist) effectively restored the reduction of IL-6 caused by ATG7 knockdown, suggesting that ATG7 can regulate IL-6 transcription through NF-κB nuclear translocation. Collectively, these findings demonstrate for the first time that ATG7 depends on NF-κB to control the transcription of IL-6 and hence increase angiogenesis but not VEGF, and they also support a new function for ATG7 outside of autophagy.

Cerebral ischemic stroke (CIS) refers to ischemic or hypoxia-induced brain necrosis or cerebral softening (Zhang et al., 2020). For patients suffering from CIS, timely and appropriate cerebral blood flow reperfusion is undoubtedly the best treatment for cerebral ischemia (Sun et al., 2022; Huang et al., 2023). However, during cerebral ischemia/reperfusion (I/R), a rapid restoration of blood flow and oxygen will worsen brain tissue damage (Zhang et al., 2022). Accumulating evidence suggests that an inflammatory response occurs during cerebral ischemia-reperfusion injury (CIRI). Inhibition of pro-inflammatory cytokine expression after stroke can reduce brain injury (Zhu et al., 2019; Han et al., 2021). Results from another study indicate that the inhibitory effect of ATG7 deletion on pro-inflammatory cytokines is achieved through NF-κB-dependent transcriptional regulation rather than inhibition of the autophagy pathway demonstrating a specific function of ATG7 independent of autophagy (Wang et al., 2018). This finding established the novel regulatory role of ATG7 in vessels associated with the inflammatory reactions during stroke and opened up fresh avenues for investigating the role of ATG7 role in vascular biology.

These studies uncover that ATG7 modulates the expression of pro-inflammatory factors such as IL-6 through NF-κB-dependent transcription, which in turn regulates cerebral angiogenesis, IS, and CIS. The pathophysiology of ischemic brain injury is enriched by this, and a novel method by which ATG7 engages in biological processes outside of the autophagy pathway is also supported. ATG7-mediated inflammatory responses of endothelial cells may be a novel target of stroke therapy. Currently, the effects of interleukins on tissue ischemia have attracted much attention, but there is still a large gap in the research on interleukins promoting wound healing and angiogenesis. Therefore, more investigation into the potential function of ATG7 as a new molecule involved in regulating the expression of pro-inflammatory proteins to encourage cerebral angiogenesis is necessary.

#### 3.1.2 ATG7 promotes angiogenesis by regulating laminin-5 through NF-κB activation

Laminin, a major component of the extracellular matrix, has been found to have a significant impact on vascular biological function (Biswas et al., 2017; Qin et al., 2017). Specifically, laminin-5 is a laminin isoform consisting of α3, β3, and γ2 chains and has been reported to induce neovascularization (He et al., 2019; Qian et al., 2021; Tohmatsu et al., 2021). In a study conducted by He et al., the molecular mechanism of ATG7 regulating cerebrovascular formation was investigated (He et al., 2019). It was found that ATG7 knockdown in HBMECs significantly reduced the expression levels of β3 and γ2 chains of laminin-5. Subsequent experiments on HBMECs treated with autophagy inhibitors revealed that the downregulation of laminin-5 expression level caused by the deletion of ATG7 remained largely unaffected (He et al., 2019). This shows that rather than autophagy, ATG7 itself is necessary for the regulatory mechanism of laminin-5 by ATG7. Exogenous laminin-5 treatment effectively rescued tube formation and migration in ATG7-deficient HBMECs (He et al., 2019), implicating that the impaired cell formation and reduced cell migration may be due to downregulation of laminin-5 expression caused by ATG7 silencing. Further investigation revealed that the phosphorylation of IκBα and IKKβ, both of which are involved in the NF-κB signaling pathway, was decreased as a result of the loss of ATG7 (He et al., 2019). Therefore, it was examined whether the modulation of laminin-5 expression by ATG7 was facilitated through the NF-κB signaling pathway. NF-κB agonists could effectively rescue ATG7 deficiency-induced downregulation of laminin-5 β3 and γ2 chains and impaired angiogenesis (He et al., 2019). ATG7 may control laminin-5 expression via the NF-κB signaling pathway, facilitating the formation of tubular structures in HBMECs. Collectively, this study proposes a novel mechanism by which the ATG7-dependent non-autophagic pathway regulates tube formation during cerebral angiogenesis. Meanwhile, this study provides a new idea for laminin-5 to regulate vascular biology.

**FIGURE 2:**
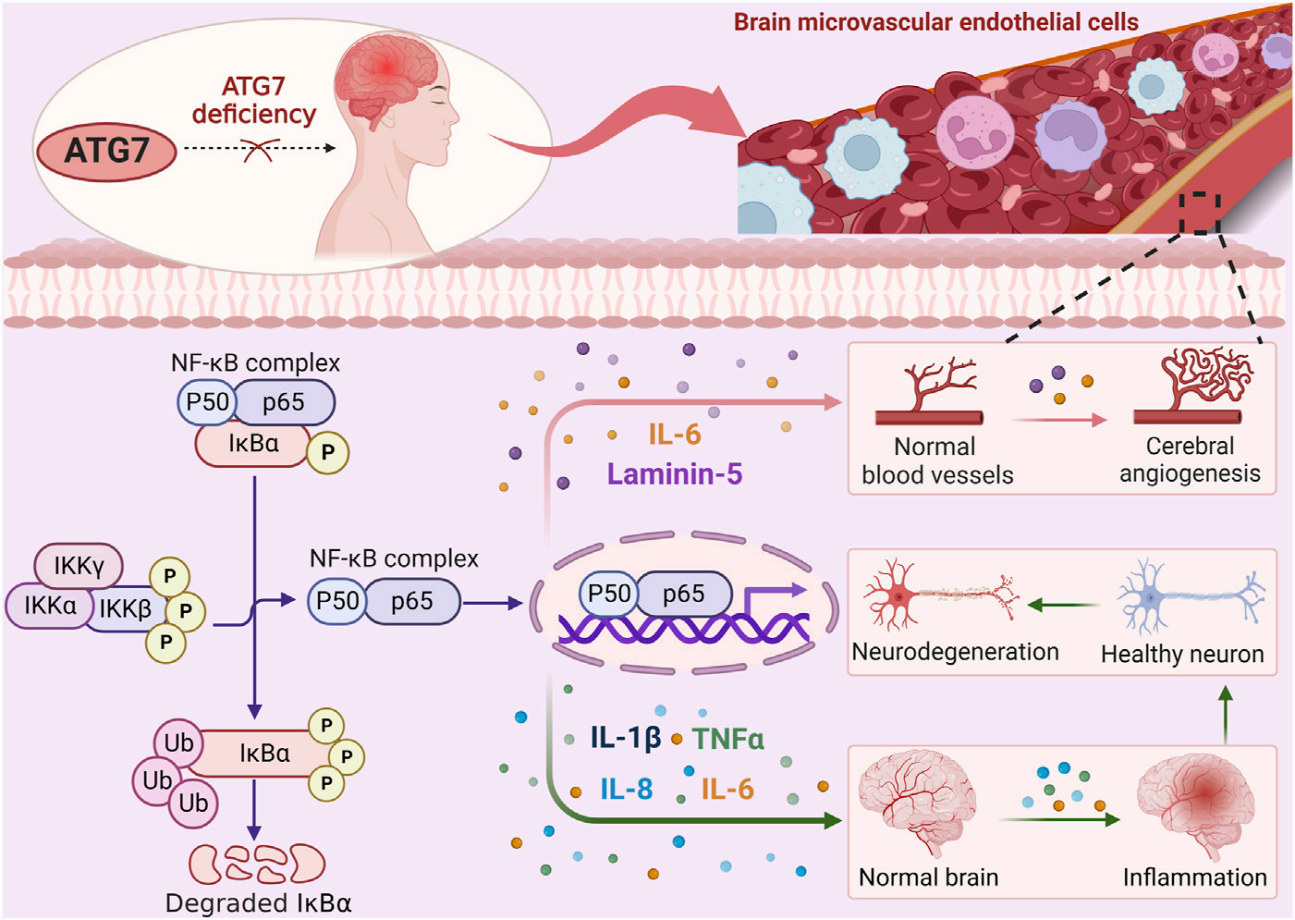
ATG7 regulates angiogenesis through NF-κB activation. ATG7 deficiency reduces IKKβ phosphorylation, thereby inhibiting IκBα phosphorylation and ubiquitination. Subsequent exposure to NF-κB nuclear localization signals is reduced, thereby attenuating the transcription and expression of reperfusion-induced pro-inflammatory cytokines such as IL-1β, TNF-α, IL-8, and IL-6, which in turn activate inflammatory responses and exacerbate brain injury. Meanwhile, the expression of IL-6 and laminin 5, which are dependent on NF-κB activation, is decreased after ATG7 knockdown, resulting in the inhibition of cerebral angiogenesis. ATG7 relies on transcriptional mechanisms independent of the autophagy pathway to regulate angiogenesis.

Based on the above results, ATG7 exhibits novel functions in transcription-dependent regulation of inflammatory responses and angiogenesis rather than just a role in the autophagy pathway, as shown in Figure 2, which exhibits the regulatory mechanisms of ATG7-dependent non-autophagic pathway, and further provides ATG7 regulation of key factors in NF-κB pathways involving in angiogenesis. IL-6 and Laminin-5 have been identified to be involved in angiogenesis through the regulation of NF-κB pathway. Notably, since IL-6 and laminin-5 have different cellular localizations, further determination of the relative contribution and interaction between IL-6 and laminin-5 in cerebral angiogenesis will help to understand the dynamic changes of cerebrovascular physiopathological states.

### 3.2 The loss of ATG7 regulates angiogenesis through STAT1 activation

An equilibrium between pro-angiogenic and anti-angiogenic factors firmly controls the process of angiogenesis (Lou et al., 2022). An experimental investigation has provided more proof that ATG7 deficiency functions as a novel angiogenic inhibitor in ischemia situations. Yao et al. (2022) used endothelial cell ATG7-specific knockout mice for femoral artery ligation, which showed the recovery of blood reperfusion in the mice was significantly impaired, angiogenesis was inhibited, and hypoxia inducible factor 1 subunit alpha (HIF1A) expression was decreased. It was also found that overexpression of ATG7 under normoxic and hypoxic conditions did not affect the level of HIF1A, whereas its knockdown reduced hypoxia-induced HIF1A expression and tube formation capacity, thus inhibiting angiogenesis (Yao et al., 2022).

**FIGURE 3:**
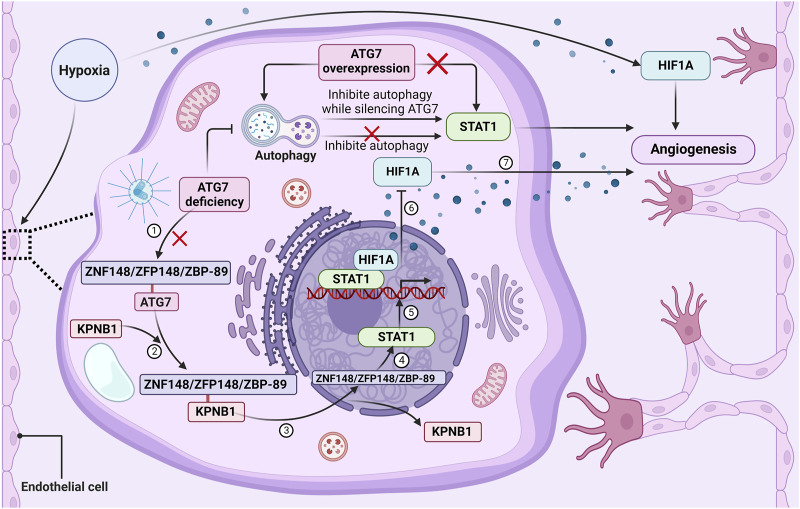
The loss of ATG7 regulates angiogenesis through STAT1 activation. ATG7 deficiency in the cytoplasm of endothelial cells will disrupt the association between ATG7 and ZNF148/ZFP148/ZBP-89. Under basal conditions, there is rarely an association between ZNF148/ZFP148/ZBP-89 and KPNB1, but the interaction between ZNF148/ZFP148/ZBP-89 and KPNB1 is significantly increased in ATG7-deficient conditions. KPNB1 transports ZNF148/ZFP148/ZBP-89 from the cytoplasm to the nucleus, resulting in ZNF148/ZFP148/ZBP-89 binding to the STAT1 gene promoter and upregulating STAT1 expression. STAT1 then binds to the HIF1A promoter and represses HIF1A expression, thereby impairing ischemia-induced angiogenesis and delaying blood flow reperfusion. Moreover, inhibition of autophagy does not affect STAT1 expression and tube formation, but silencing ATG7 while inhibiting autophagy significantly upregulates STAT1 expression and tube formation, suggesting that ATG7 deficiency upregulates STAT1 expression and inhibits blood vessel formation in an autophagy-independent manner. ATG7 overexpression activates autophagy but does not affect ZNF148/ZFP148/ZBP-89 nuclear translocation, STAT1 expression, and tube formation under both normoxic and hypoxic conditions. Lack of ATG7 inhibits angiogenesis by suppressing HIF1A expression at the transcriptional level through upregulation of STAT1 independently of autophagy under ischemic conditions.

Mechanistically, a group of transcription factors including signal transducer and activator of transcription 1(STAT1), signal transducer and activator of transcription 3 (STAT3), NF-κB, interferon regulatory factor 9 (IRF9), nuclear respiratory factor 1 (NRF1), and BCL2 associated transcription factor 1 (BCLAF1) can bind to the HIF1A gene promoter and regulate HIF1A expression (Gerber and Pober, 2008; Wang et al., 2016; Park et al., 2017; Shao et al., 2020; Li Z. L. et al., 2021b; He et al., 2022; Niu et al., 2022). Further analysis of the expression of these six transcription factors during ATG7 silencing revealed that STAT1 was the most abundantly expressed in human umbilical vein endothelial cells (HUVECs), followed by IRF9, while the expression of other transcription factors did not change significantly (Yao et al., 2022). The STAT1 gene was also discovered to drastically reduce the production of HIF1A brought on by ATG7 loss (Yao et al., 2022). Adding to existing knowledge of the role of ATG7 in angiogenesis, inhibition of autophagy was noted to not increase the level of STAT1 expression and have no effect on HUVEC tube formation, whereas inhibiting autophagy while silencing ATG7 significantly upregulated STAT1 expression and tube formation. However, overexpression of ATG7 promoted autophagy and had no effect on STAT1 levels (Yao et al., 2022). Furthermore, it has been discovered that ATG7 plays a role in controlling the expression of STAT1 by interacting with ZNF148/ZFP148/ZBP-89 (zinc finger protein 148), a transcription factor required for constitutive expression of STAT1. Specifically, the lack of ATG7 in the cytoplasm disrupted the association between ATG7 and zinc finger protein 148 and enhanced the binding of zinc finger protein 148 to karyopherin subunit beta 1 (KPNB1), thus promoting the nuclear translocation of zinc finger protein 148 and enhancing the expression of STAT1 (Yao et al., 2022). These results provide evidence indicating a role for ATG7 in angiogenesis, as shown in Figure 3, ATG7 deficiency impairs post-ischemic angiogenesis by upregulating STAT1 expression in an autophagy-independent manner, thereby repressing HIF1A expression at the transcriptional level.

Together, this study shows that ATG7 absence can be a significant angiogenesis inhibitor. The level of STAT1 is upregulated by ATG7 deficiency through the interaction between ATG7 and zinc finger protein 148 and independently of the autophagy pathway. Subsequently, STAT1 suppresses HIF1A expression by binding to the HIF1A promoter, thereby inhibiting ischemia-induced angiogenesis and delaying blood flow reperfusion (Yao et al., 2022). Given the important roles of angiogenesis in the regulation of tumor cell proliferation and survival, targeting ATG7 offers a novel approach to improving antitumor therapy. Meanwhile, inhibition of STAT1 by either genetic or pharmacological means is suggested to counteract the inhibitory effect of ATG7 deficiency on ischemia-induced angiogenesis. This implies that STAT1 inhibition may also be a useful therapeutic strategy for the management of ischemic cardiovascular illness, which may ultimately result in improved clinical care for patients with either ischemic cardiovascular disease or cancer.

## 4 Discussion and conclusion

Autophagy is a tightly regulated process mediated by the concerted actions of ATG proteins. Studies have shown that autophagy is closely related to angiogenesis. This review has added some examples of non-autophagic roles of ATG proteins, and provided evidences on ATG7-dependent autophagic angiogenesis. Furthermore, several studies have indicated that ATG proteins, especially ATG7, can regulate angiogenesis through both autophagic and non-autophagic pathway. ATG7 facilitates the maturation of the autophagosomes, however, non-autophagic functions of core autophagy proteins including ATG7 have been reported. Atg7 is closely associated in angiogenesis, prompting consideration that non-autophagy functions of ATG7 may regulate angiogenesis. Additionally, ATG7 is found to regulate vascular cell growth, migration, and tube formation through a non-autophagic pathway but not an autophagy pathway. This could be interesting for future studies in which researchers explore new functions of known proteins. However, the experimental results of the non-autophagy function of ATG7 may not reflect the whole view of it. Artificial errors could be included by using current cellular and biological research methods. Noteworthily, this review provides evidence that the effects of ATG7 can extend beyond its autophagy suggesting that ATG7 regulates angiogenesis through non-autophagic pathways. Interestingly, the autophagy-independent function of ATG7 was also previously reported by Lee et al. (2012), who found that ATG7, independent of its E1-like enzymatic activity, could bind to the tumor suppressor p53 to regulate the transcription of the gene encoding the cell cycle inhibitor p21CDKN1A. Moreover, Hwang et al. (2012) found that the direct antiviral activity of IFNγ against MNV in macrophages requires ATG5-ATG12, ATG7, and ATG16L1, but does not require the induction of autophagy, the degradative activity of lysosomal proteases, and the fusion of autophagosomes with lysosomes or ATG4B (Hwang et al., 2012). ATG5, ATG7, ATG4B, and LC3 participate in the polarized secretion of lysosomal contents into the extracellular space by directing lysosomes to fuse with the plasma membrane (DeSelm et al., 2011). Additionally, other non-autophagic functions of autophagy proteins have been reported. As shown in Supplementary Table S1, autophagy proteins may play non-autophagic functions in transcription (Lee et al., 2012), cell survival and apoptosis (Yousefi et al., 2006; Wirawan et al., 2010), cellular transport (Ogura et al., 2010), protein secretion (Ishibashi et al., 2012), cell signaling and membrane reorganization (Baisamy et al., 2009; Starr et al., 2012). Based on it, there’s a reasonable prospect that the regulation of angiogenesis by ATG7 can depend on non-autophagy pathways.

**FIGURE 4:**
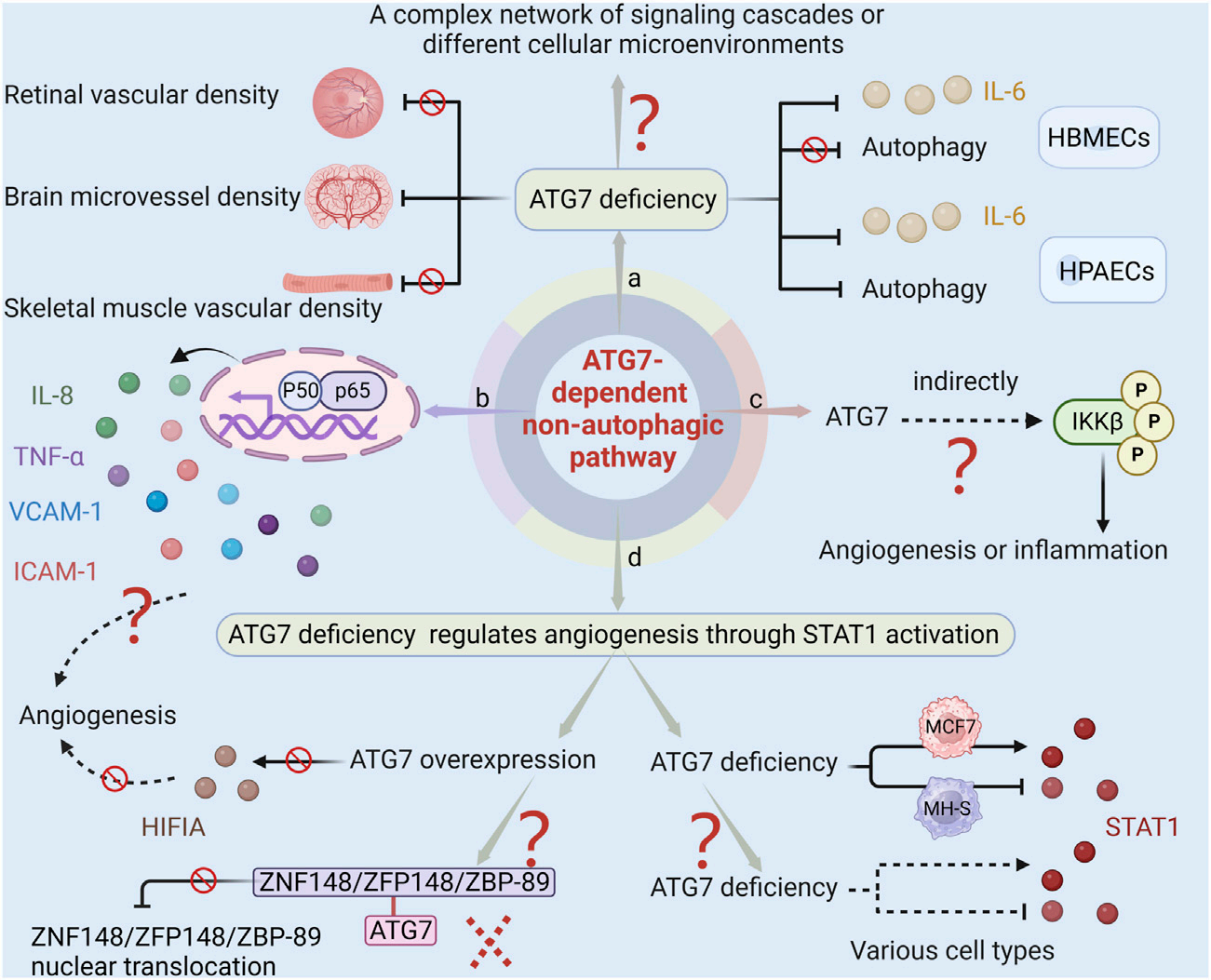
The current issues regarding ATG7-dependent non-autophagic pathway. (a) ATG7 deficiency inhibits both lipopolysaccharide-induced IL-6 production and autophagy in HPAECs. However, in HBMECs, ATG7 deficiency impacts IL-6 production without affecting the process of autophagy. Moreover, EC-specific ATG7 knockout significantly reduces brain microvessel density but does not affect skeletal muscle or retinal vascular density. These phenomena may be caused by ATG7-induced complex networks of signaling cascades or by different cellular microenvironments. (b) Given that IL-8, TNF-α, VCAM-1, and ICAM-1 in the NF-κB pathway play a role in angiogenesis, and ATG7 deletion inhibits angiogenesis by regulating NF-κB activity. It can therefore be conjectured that other cytokines may also be involved in the regulation of neovascularization by ATG7. (c) Since ATG7 lacks a kinase domain, and some protein kinases such as NIK, AKT, MEKK, COT/TPL-2, and TAK1 can phosphorylate IKK, it is speculated that ATG7 indirectly regulates the phosphorylation of IKKβ, which subsequently affects the release of inflammatory cytokines and angiogenesis. (d) There are two unanswered questions regarding the study of ATG7 deficiency activating STAT1 to regulate angiogenesis. One is that ATG7 overexpression has no significant effect on HIFIA levels and tube formation. This could be because ATG7 overexpression does not increase the association between ZNF148/ZFP148/ZBP-89 and ATG7, so it cannot reduce the nuclear translocation of ZNF148/ZFP148/ZBP-89, nor can it reduce STAT1 expression and promote HIF1A expression. The other is that ATG7 deficiency inhibits STAT1 activation in MH-S and induces STAT1 expression in MCF7. Whether the regulation of STAT1 by ATG7 in various cells is consistent with the observation that ATG7 deficiency promotes STAT1 expression in ECs remains unclear.

The study of the ATG7-dependent non-autophagic pathway is still in its infancy, with many previously unexplored questions remaining unanswered, as shown in Figure 4, which exhibits the current issues regarding the regulatory mechanism of ATG7-dependent non-autophagic pathways. In multiple models of angiogenesis, ATG7 deficiency has been shown to impact several angiogenic factors such as IL-6, laminin-5, and HIF1A, without affecting the process of autophagy. However, Shadab et al. (2020) discovered that in human pulmonary artery endothelial cells (HPAECs), ATG7 knockout not only inhibited lipopolysaccharide-induced IL-6 production but also significantly inhibited autophagy. This finding differs from the previous one that ATG7 knockout inhibits IL-6 production in an autophagy-independent manner. Meanwhile, EC-specific ATG7 knockout significantly reduces brain microvessel density (Zhuang et al., 2017) but does not affect skeletal muscle or retinal vascular density under normoxic conditions (Torisu et al., 2013). These phenomena may be caused by ATG7-induced a complex network of signaling cascades or by different cellular microenvironments.

Additional research is required to ascertain whether, in addition to the previously documented IL-6, laminin-5, and HIF1A, other angiogenic factors also contribute to neovascularization under the impact of ATG7. NF-κB is a crucial molecule that regulates the expression and function of multiple genes. It plays a significant role in various physiological processes such as vascular cell growth, migration, tube formation, and inflammatory responses. Earlier research has indicated that genes IL-8, TNF-α, vascular cell adhesion molecule 1 (VCAM-1), and intercellular adhesion molecule 1 (ICAM-1) in the NF-κB pathway play a role in angiogenesis (Yoshida et al., 1998; Bakshi et al., 2022; Lian et al., 2022). The deletion of ATG7 has been found to closely regulate NF-κB activity, which in turn leads to the inhibition of angiogenesis. This suggests that ATG7 may function as a gene that regulates both inflammation and angiogenesis. While it has been demonstrated that ATG7 plays a role in regulating cerebral angiogenesis through the NF-κB-mediated IL-6 and laminin-5 pathways, it is important to note that other cytokines may also be involved in the regulation of neovascularization by ATG7.

IKKβ is the major kinase involved in the activation of the NF-κB signaling pathway triggered by proinflammatory factors (Yu et al., 2022). In response to external stimulation, IKKβ phosphorylates and degrades IκBα, and the dissociative NF-κB subsequently translocates into the nucleus and exposes the nuclear localization signals on the p65/p50 heterodimer, which leads to pro-inflammatory factors transcription (Siracusa et al., 2021). ATG7 interacts with p-IκBα and regulates the NF-κB signaling pathway during *Klebsiella pneumoniae* infection (Ye et al., 2015). ATG7 downregulation prevents IKKβ phosphorylation, which in turn results in less NF-κB activation (Wang et al., 2018). Despite intensive study of the NF-κB signaling pathway, the precise molecular mechanisms governing ATG7’s control of IKKβ phosphorylation and consequent NF-κB activation are still unknown. Based on the fact that ATG7 lacks a kinase domain, while some protein kinases such as mitogen-activated protein kinase kinase kinase 14 (NIK), protein kinase B (AKT), MAPK/ERK kinase kinase family protein (MEKK), mitogen-activated protein kinase kinase kinase 8 (COT/TPL-2), and TGF-beta activated kinase 1 (TAK1) can phosphorylate IKK (Karin and Ben-Neriah, 2000; Hiscott et al., 2001), it is conjectured that ATG7 does not directly regulate the phosphorylation of IKKβ, but indirectly influences the phosphorylation of IKKβ and the activation of NF-κB. Further research on the regulation of the ATG7-dependent NF-κB pathway, specifically in regards to its impact on inflammatory cytokines and angiogenesis, can be enriched by investigating the role of ATG7 in regulating IKKβ phosphorylation through targeting protein kinases. This research holds significant potential for advancing our understanding of the underlying mechanisms involved in these processes.

Future research on ATG7’s suppression of ischemia-induced angiogenesis through STAT1 activation is called for because there are still some unresolved issues. For example, ATG7 deletion reduces hypoxia-induced HIF1A expression and tube formation, but overexpression of ATG7 has no significant effect on the level of HIFIA and tube formation ability. The reason for this phenomenon may be that although overexpression of ATG7 does lead to an increase in its level, it does not significantly enhance the correlation between zinc finger protein 148 and ATG7, thereby failing to affect the nuclear translocation of zinc finger protein 148, and the subsequent expression of STAT1 and HIF1A in the nucleus has not changed significantly. Future studies are needed to address how overexpressing ATG7 alters the relationship between ATG7 and zinc finger protein 148 to facilitate angiogenesis. Another issue worthy of attention is that the regulation of STAT1 by ATG7 may vary greatly across cell types. In mouse alveolar macrophage cell lines, ATG7 deficiency inhibits STAT1 activation, whereas in human breast cancer MCF7 cells, ATG7 deficiency leads to an increase in the expression of STAT1 protein (Ambjørn et al., 2013; Li et al., 2015). Various cell types are involved in angiogenesis, including vascular smooth muscle cells, pericytes, macrophages, etc. Whether the regulation of ATG7 on STAT1 in these cells is consistent with that observed in endothelial cells requires future clarification.

Research on the role and mechanism of ATG7 in angiogenesis is still in its infancy. The regulatory relationship between ATG7 and intracellular factors, as well as the molecular mechanism of vascular remodeling regulation by ATG7, are not yet fully understood. In addition, studies on the effects of ATG7 on vascular endothelial cells have mostly concentrated on cellular-level study, with few data on the pathomorphological changes to vascular tissue brought on by ATG7 at the level of the entire tissue. Therefore, in-depth studies on the mechanism of ATG7 regulation of angiogenesis are needed to discover more effective therapeutic targets for vascular disease treatment. Given the complex interactions between ATG7-mediated autophagy-dependent and autophagy-independent pathways, more detailed analysis of molecular biology assays and *in vivo* pathophysiologic experiments are necessary for the dissection of the exact roles of each signaling pathway in different angiogenic models or different pathological conditions. Additionally, it is unclear whether ATG7 controls other biological processes via non-autophagic channels in addition to controlling angiogenesis or whether there are other autophagy-related proteins regulating biological functions via non-autophagic pathways in addition to ATG7. These growing, yet unexplored, research topics deserve special attention in the future.

## Figures and Tables

**Table:** 

**ATG7**	autophagy related 7
**ATGs**	autophagy-related genes
**LC3**	microtubule-associated protein 1 light chain 3
**ATG4**	autophagy related 4
**ATG5**	autophagy related 5
**ATG8**	autophagy related 8
**ATG10**	autophagy related 10
**ATG12**	autophagy related 12
**ATG16**	autophagy related 16
**NTD**	N-terminal domain
**CTD**	C-terminal domain
**ECTD**	extreme C-terminal ATG7-specific domain
**AD**	homodimeric adenylation domain
**PE**	phosphatidylethanolamine
**CNS**	central nervous system
**IS**	ischemic stroke
**HBMECs**	human brain microvascular endothelial cells
**IL-6**	interleukin-6
**NF-κB**	nuclear factor-kappa B
**CIS**	cerebral ischemic stroke
**I/R**	ischemia/reperfusion
**CIRI**	cerebral ischemia-reperfusion injury
**IL-1β**	interleukin-1beta
**IL-8**	interleukin-8
**TNF-α**	tumor necrosis factor alpha
**STAT1**	signal transducer and activator of transcription 1
**STAT3**	signal transducer and activator of transcription 3
**IRF9**	interferon regulatory factor 9
**NRF1**	nuclear respiratory factor 1
**BCLAF1**	BCL2 associated transcription factor 1
**HUVECs**	human umbilical vein endothelial cells
**KPNB1**	karyopherin subunit beta 1
**HPAECs**	human pulmonary artery endothelial cells
**VCAM-1**	vascular cell adhesion molecule 1
**ICAM-1**	intercellular adhesion molecule 1
**NIK**	mitogen-activated protein kinase kinase kinase 14
**AKT**	protein kinase B
**MEKK**	MAPK/ERK kinase kinase family protein
**COT/TPL-2**	mitogen-activated protein kinase kinase kinase 8
**TAK1**	TGF-beta activated kinase 1
**MCF7**	human breast cancer cell line
